# Effects of Phytogenically Synthesized Bimetallic Ag/ZnO Nanomaterials and Nitrogen-Based Fertilizers on Biochemical and Yield Attributes of Two Wheat Varieties

**DOI:** 10.3390/nano12172894

**Published:** 2022-08-23

**Authors:** Maria Ehsan, Naveed Iqbal Raja, Zia Ur Rehman Mashwani, Efat Zohra, Fozia Abasi, Muhammad Ikram, Nilofar Mustafa, Feroza Hamid Wattoo, Jarosław Proćków, José Manuel Pérez de la Lastra

**Affiliations:** 1 Department of Botany, PMAS-Arid Agriculture University Rawalpindi, Rawalpindi 46000, Pakistan; 2 University Institute of Biochemistry and Biotechnology, PMAS-Arid Agriculture University Rawalpindi, Rawalpindi 46000, Pakistan; 3 Department of Plant Biology, Institute of Environmental Biology, Wrocław University of Environmental and Life Sciences, Kożuchowska 5b, 51-631 Wrocław, Poland; 4 Biotechnology of Macromolecules Research Group, Instituto de Productos Naturales y Agrobiología (IPNA CSIC), 3-38206 San Cristóbal de la Laguna, Spain

**Keywords:** nanobiotechnology, plant physiology, crop production, agriculture, fertilizers, green synthesis

## Abstract

Wheat is the most important staple food worldwide, but wheat cultivation faces challenges from high food demand. Fertilizers are already in use to cope with the demand; however, more unconventional techniques may be required to enhance the efficiency of wheat cultivation. Nanotechnology offers one potential technique for improving plant growth and production by providing stimulating agents to the crop. In this study, plant-derived Ag/ZnO nanomaterials were characterized using UV-Vis spectroscopy, SEM, EDX, FTIR, and XRD methods. Various concentrations of phytogenically synthesized Ag/ZnO nanomaterials (20, 40, 60, and 80 ppm) and nitrogen-based fertilizers (urea and ammonium sulphate 50 and 100 mg/L) were applied to wheat varieties (Galaxy-13 and Pak-13). The results obtained from this research showed that application of 60 ppm Ag/ZnO nanomaterials with nitrogenous fertilizers (50 and 100 mg/L) were more effective in improving biochemistry and increasing yield of wheat plants by reducing enzymatic and non-enzymatic antioxidants (proline content, soluble sugar content, malondialdehyde, total phenolic content, total flavonoid content, superoxide dismutase, peroxidase, and catalase); and significantly increasing the protein content, number of grains per pot, spike length, 100-grain weight, grain yield per pot, and harvest index of both wheat varieties, compared to untreated plants. These findings allow us to propose Ag/ZnO nanomaterial formulation as a promising growth- and productivity-improvement strategy for wheat cultivation.

## 1. Introduction 

Wheat (*Triticum aestivum* L.) provides over 20% of worldwide food energy and protein as a staple cereal and is the most essential staple food crop, providing carbohydrates and protein to more than 50% of the world’s population [1]. However, wheat grain yields have remained stable in many wheat-growing areas around the globe. In Pakistan, wheat is the most important food crop, exceeding all other crops in measures of land usage and productivity, with a high nutritional demand for nitrogen, predominantly at the propagative stage [2]. For wheat cultivation, higher concentrations of nitrogenous fertilizers are required to boost grain production and increase protein content. These fertilizers are the main agrarian input for grain production, despite the ecological problems caused by their use [3]. However, any reduction in the use of nitrogenous fertilizers leads to lower crop yields and possible scarcity and hunger, while increased use of nitrogenous fertilizers may lead to serious losses of nitrogen, freshwater eutrophication, atmospheric contamination, and increased annual emissions of anthropogenically produced carbon dioxide [4]. Optimizing the amount of nitrogenous fertilizer used is the key to the best use of such fertilizers [5]. We must consider the three critical issues of food safety, ecological quality, and climate variation, when assessing advances in nitrogen utilization, as it relates to future crop production worldwide [6]. 

The world’s greatest challenges in such areas as agriculture, biodiversity, energy, health, and water can all be addressed by bio-nanotechnology. Bio-nanotechnology deals with nano-sized materials (1 to 00 nanometers) [7]. Due to their smaller size, nanomaterials (NMs) can easily penetrate the soil, aiding the advantageous microbe population responsible for nutrition availability to plants via the mineralization of numerous essential minerals [8]. Some nanomaterials are already used in the agrarian field for the improvement of growth and production of various crops [9]. Nanomaterials have been shown to improve agro-morphological and physico-biochemical attributes in crops such as beans and maize [10]. The use of nanomaterials as fertilizers can boost yields without any harmful effects on soil [11]. 

Plant-derived nanomaterials are stimulating fertilizers that can be complemented with single or multiple nutrients, to boost plant growth and development more effectively than traditional fertilizer alone [12]. Moreover, nanomaterials are ecologically advantageous because they increases efficiency in crop production, resulting in lower input costs of labor and waste. Nanomaterials are varied collections of metals and perform a lively role in the process of crop growth [13]. Recently, researchers have reported the effects of nanomaterials on both plant development and subsequent storage from a nutritional perspective. Both metal- and metal oxide-based nanomaterials have been shown to affect plant metabolism [14]. 

The combination of two metals as bimetallic nanomaterials—synthesized phytogenically with synergistic effects—results in certain new features and increased functionality. A combination of bi-functionality effects, lattice stresses, and electronic effects results in synergism [15]. In recent years, bimetallic nanomaterials have gained increasing attention because of their applications in a wide range of fields. Such materials and are of value due to their unique properties, which are distinct from monometallic nanomaterials [16]. When the existing three approaches to synthesis are compared, the biogenic approach is shown to be more effective and less expensive than the physical or the chemical approach for the synthesis of nanomaterials, paving the way for an forthcoming era of bio-nanotechnology. The use of nanomaterials reduces the requirement for fertilizers in fields, with enhanced effectiveness and greater control of impacts upon the ecosystem [17]. 

In recent years, silver-based nanomaterials have been manufactured for a wide array of industrial applications. Ag-based NPs have been used in farming for the improvement of crops. The effect of Ag-based nanomaterials on the morpho-physiology of plants depends on the morphology of the nanomaterials used [18]. Various studies have shown that silver-based nanomaterials at an optimum concentration can play a significant role in increasing seed germination, photosynthetic quantum efficacy, and chlorophyll content; in enhancing the efficiency of water, mineral, and fertilizer use; and in plant growth [19,20]. The effects of biologically synthesized Ag-based nanomaterials on germination of seeds with reduced phenol content, and on the induction of carbohydrate–protein synthesis in *Bacopa monnieri* have also been reported in the literature [21]. Better growth profiles and improvements in biochemical parameters such as antioxidant enzymes, carbohydrates, chlorophyll, and protein content have also been achieved by the application of Ag-based nanomaterials to the common bean and *Brassica juncea* [22].

Zinc oxide-based nanomaterials have the potential to improve the growth and yield of nutritional crops. The optimum concentration of zinc oxide-based materials on a nanoscale promotes germination and growth of seeds, resulting in higher seedling vigor index values, and enhanced growth of root and stem in wheat crops. ZnO-based nanomaterials in colloidal solution forms can be used as enrichers, and already play an important role in the agrarian field [23,24]. As nutrients, nano-sized enrichers are more important to the plant than fertilizers as they not only provide nutrients but also restore the organic qualities of the soil, reversing the adverse effects of chemical fertilizer [25]. When seeds of wheat plants treated with metal-based nanomaterials germinate in the soil, growth increases, and production rate increases by 20–25%. Hence, the use of metal and metal oxide-based nanomaterials should increase growth and production rates of crops such as wheat and flax [26]. The nutritional requirements of the world are daily increasing while the yield of staple food crops remains stable; the time has come, therefore, for the commercialization of metal- and metal oxide-based nanomaterials for the purposes of sustainable agriculture [27]. To this end, keeping in view the synergism of bimetals and the requirement of nitrogen-based fertilizers for crops, the present research has been designed to study the role of plant-derived synthesized bimetallic Ag/ZnO nanomaterials and nitrogen-based fertilizers on the biochemical and yield attributes of wheat varieties. In doing so, we may help introduce an unconventional technique to improve crop production and so help to meet the worldwide demand for food.

## 2. Materials and Methods

**Figure 1:**
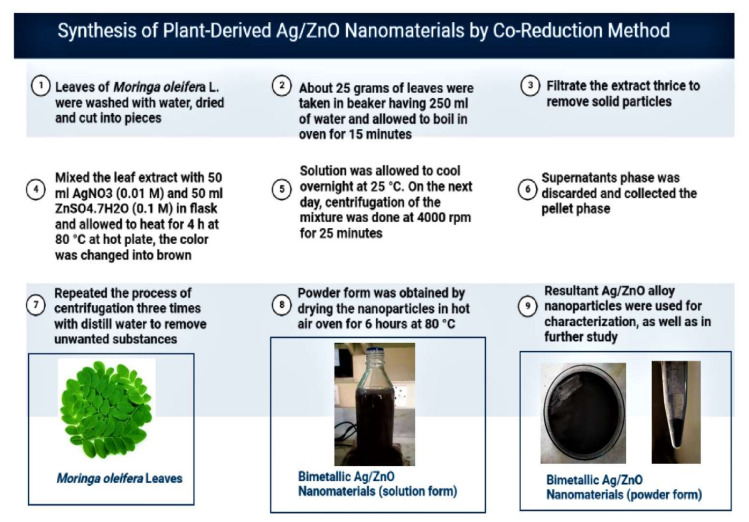
Method of preparation of nanomaterials.

**Figure 2:**
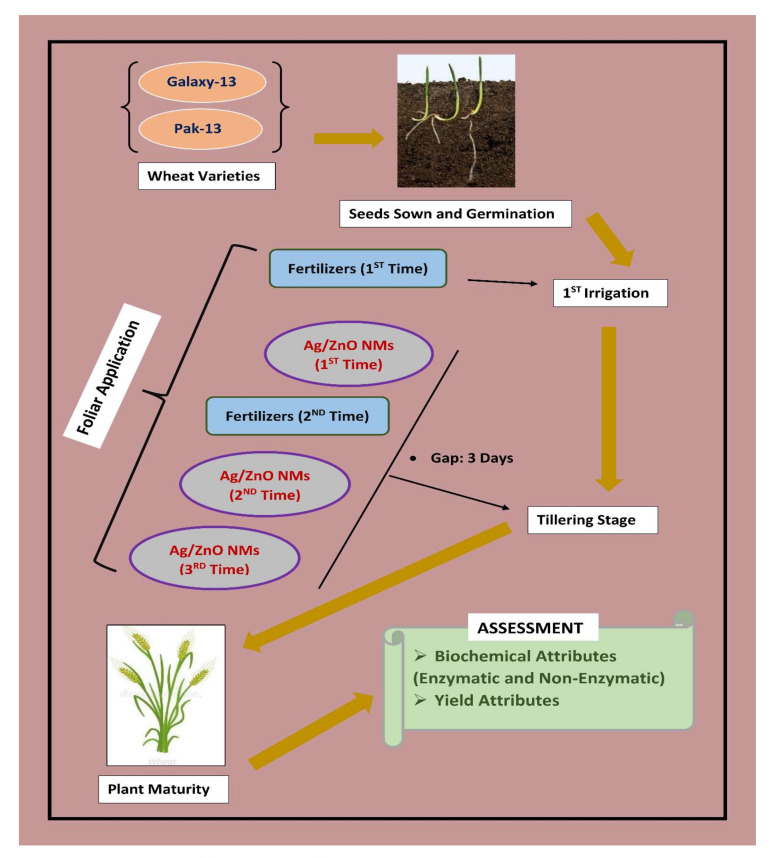
Schematic illustration of research study.

Bimetallic Ag/ZnO nanomaterials were phytogenically synthesized using leaf extracts of *Moringa oleifera* L. in a solution of silver nitrate and zinc sulfate salts following the co-reduction method of Sorbium et al. [28] with certain modifications. A detailed account of the synthesis method is presented in Figure 1. Two varieties of bread wheat widely cultivated in Pakistan were selected (Galaxy-13 and Pak-13). Various concentrations of two nitrogen-based fertilizers (urea and ammonium sulfate) and bimetallic Ag/ZnO nanomaterials were formulated. Figure 2 shows a schematic illustration of the whole experiment. Fertilizers were initially applied at the time of the first irrigation, then again at the tillering stage three days after nanomaterials application (1st time) and/or three days before nanomaterials application (2nd time). The experiment was conducted in the greenhouse in the research area of the Botany Department, PMAS-AAUR, Pakistan. Table 1 sets out the treatments used in the experiment and gives details of the different concentrations of fertilizers and plant-derived Ag/ZnO NMs used, with T_0_ considered as control. Subsequently, for each treatment, measures of biochemical (non-enzymatic contents and enzymatic activity) and yield attributes were taken from plant samples. To determine the absorbance wavelength for evaluation of biochemical contents, a U-2900UV/VIS spectrophotometer 200 V; model no. 2JI-0004 was used.

### 2.1. Characterization Techniques

The phytogenically prepared bimetallic Ag/ZnO nanomaterials were added to test tubes along with distilled water and sonicated for 15 min. Then, the UV–visible spectrum of the solution was examined by the use of UV–visible spectroscopy. Scanning electron microscopy was used for structural analysis of Ag/ZnO nanomaterials. For this purpose, a minute quantity of sample was placed on the grid (carbon coated) to make the thin layered film and dried for 10 min under a mercury lamp. Elemental analysis of this sample was then carried out through energy-dispersive X-ray spectroscopy. In addition, Fourier-transform infrared spectroscopy (FTIR) analysis was performed using FTIR Bruker Alpha II by placing a minute quantity of Ag/ZnO NPs powder on an ATR crystal spot in transmission wavelength mode, using OPUS software. An X-Ray diffraction (XRD) study was carried out using X-ray diffraction spectroscopy by analyzing samples from 5 to 50 at 2 angles. An average size of NMs was recorded by the Debye–Scherrer equation: D = kλ/βcosθ) [28,29].

### 2.2. Biochemical Contents Assays

The method described by Bates et al. [30] was followed for the determination of proline content (µg/mL). Fresh leaves of 0.2 g were crushed in 3 percent sulfosalicylic acid (2 mL), and filtrates (2 mL) were taken in test tubes. Ninhydrin reagent (2 mL) and glacial acetic acid (2 mL) were then added and allowed to boil in a water bath till color developed, and the reaction was stopped by placing the test tubes in ice. Toluene (4 mL) was then added, and solutions were properly mixed by shaking until an upper colored layer was observed. This layer was then separated into another test tube for each sample. Absorbance was observed at a wavelength of 520 nm and proline content was calculated by the formula:

$$\mathrm{Total}\;\mathrm{Proline}=\frac{\left(\mathrm{Sample}\;\mathrm{Absorbance}\times \mathrm{Dilution}\;\mathrm{Factor}\times K\;\mathrm{value}\right)}{\mathrm{Fresh}\;\mathrm{Weight}}$$

The phenolic sulfuric acid method used by Dubois et al. [31] was followed for the determination of soluble sugar content (µg/mL). Fresh leaves of 0.5 g were crushed in 80 percent ethanol (10 mL) and allowed to heat in a water bath at 80 °C for an hour. Extracts (0.5 mL) were taken in another set of test tubes, 18 percent phenol (1 mL) was added, and mixtures were incubated for an hour at 25 °C. Then, after the addition of sulfuric acid (2.5 mL), mixtures were properly mixed, and absorbance of each sample was observed at a wavelength of 490 nm. Calculations were then made using the formula:

$$\mathrm{Soluble}\;\mathrm{Sugar}\;\mathrm{Content}=\frac{\left(\mathrm{Sample}\;\mathrm{Absorbance}\times \mathrm{Dilution}\;\mathrm{Factor}\times K\;\mathrm{value}\right)}{\mathrm{Fresh}\;\mathrm{Weight}}$$

The method used by Lowry et al. [32] was followed for the determination of protein content (µg/mL). Fresh leaves of 0.5 g were crushed in phosphate buffer and filtrates (0.5 mL) were taken. Distilled water (0.5 mL), and bio-rad dye reagent (3 mL) were then added. Then, the solutions were properly vortexed, and the absorbance of each sample was observed at a wavelength of 595 nm.

### 2.3. Non-Enzymatic Antioxidant Activity Assays

The thiobarbituric acid (TBA) test used by Bailly et al. [33] was followed for the determination of malondialdehyde content (MDA) expressing lipid peroxidation. Trichloroacetic acid (TCA) of 0.1% was used for homogenization of fresh leaves and centrifuged for 15 min at 16,000 rpm. Then, 3 mL of 0.5% TBA and 1 mL of 5% TCA were added to the supernatant, and mixtures were heated for 30 min at 95 °C. Mixtures were then cooled instantly in an ice box and centrifuged for 10 min at 16,000 rpm. The absorbance of each sample was observed at wavelengths of 532 nm and 600 nm. Values at 600 nm were then subtracted from those of 532 nm. MDA content was expressed in micrograms per milligram of fresh weight, while the extinction coefficient was 155 mM^−1^cm^−1^.

By following the method of Giri et al. [34], extracts were prepared for the determination of total phenolic content (TPC-µg/mg dry weight) and total flavonoid content (TFC-µg/mg dry weight). Dried leaves of 1 g were placed in 80 percent methanol (10 mL), vortexed and centrifuged for 10 min at 8000 rpm. Supernatants were either used immediately or stored at 4 °C for TPC and TFC analysis. The method used by Velioglu et al. [35] was followed for the determination of TPC. The Folin–Ciocalteu reagent (10 folds) was diluted with distilled water and 750 µL was mixed with plant extract (100 µL). After 5 min, 5 percent sodium bicarbonate was added (750 µL) and the mixture was set aside for half an hour. Absorbance of each sample was observed at a wavelength of 725 nm. The method used by Chang et al. [36] was followed for the determination of TFC. To 10 mL of 80 percent ethanol, 10mg of quercetin was added, then diluted up to 25, 50, 75 and 100 µm/mL. Then, plant extract (100 µL), 10 percent aluminum chloride (100 µL), 1 M potassium acetate (100 µL), 95 percent ethanol (1.5 mL) and distilled water (2.8 mL) were mixed with each diluted solution (400 µL). Mixtures were kept at 25 °C for 30 min and absorbance was observed at a wavelength of 415 nm.

### 2.4. Antioxidant Enzymatic Activity Assays

The extracts of antioxidant enzymes such as superoxide dismutase (SOD), peroxidase (POD) and catalase activity (CAT) were prepared by the method used by Nayyar and Gupta [37]. Fresh leaves of 0.1 g were prepared in extraction buffer (10 mL), sonicated three times, and centrifuged for 10 min at 8000 rpm. Supernatants were then collected. Extracts were either used immediately or stored at 4 °C for anti-oxidative enzyme analysis. For the calculation of these three enzymes’ activity, the Lambert–Beer Law was applied thus:

A=εLC

where, A is absorbance, ε is the extinction coefficient (6.39 Mm^−1^cm^−1^), L is the length of the wall (25 cm) and C is the concentration of enzymes (expressed in nM/min/mg FW). For the determination of SOD activity, the method used by Perez [38] was followed. The reaction mixture of 1 mL consisted of 130 mM methionine (100 µL), 1mM EDTA (100 µL), enzyme extract (300 µL), 0.075 mM nitro blue tetrazolium (100 µL), phosphate buffer (390 µL), and 0.02 mM riboflavin (10 µL), while the blank mixture included phosphate buffer instead of plant extract. Under fluorescent light, both reactions were allowed to continue for 7 min and optical density was observed at a wavelength of 560 nm. For the determination of LOD, the method used by Lagrimini [39] was followed. The reaction mixture of 1 mL consisted of distilled water (500 µL), enzyme extract (100 µL), 100 mM guaiacol (100 µL), 27.5 mM hydrogen peroxide (100 µL), and phosphate buffer (200 µL), while the blank mixture included phosphate buffer instead of plant extract. Under fluorescent light, both reactions were allowed to continue for 7 min and optical density was observed at a wavelength of 470 nm. For the determination of CAT, the method used by Aebi [40] was followed. The reaction mixture of 1 mL consisted of distilled water (450 µL), enzyme extract (250 µL), 27.5 mM hydrogen peroxide (100 µL), and phosphate buffer (200 µL), while the blank mixture included phosphate buffer instead of plant extract. Under fluorescent light, both reactions were allowed to continue for 7 min and optical density was observed at a wavelength of 470 nm.

### 2.5. Yield Parameters

At full maturity, when plants were ready to harvest, yield attributes were recorded by collecting data for various parameters, i.e., the number of grains per pot, spike length (cm), 100-grain weight (gm), and grain yield per pot (in gm). Values for the harvest index were calculated using the following formula, where biomass per plant equals the dry weight of each plant (in gm).

$$Harvest\;Index\;\left(\%\right)=\frac{Grain\;yield\;per\;pot\;}{Biomass\;per\;plant}\times 100$$

### 2.6. Statistical Analysis

In this study, each treatment consisted of three replicates, while results were interpreted in terms of mean and standard errors (±SE). Statistical analysis was carried out by one-way analysis of variance using SPSS software (16.1) with statistical significance (*p* ≤ 0.05). The method of Latin square design was also applied to study significant differences among treatments. Treatments which showed the best results were compared with the control in term of percentages.

## 3. Results

### 3.1. Characterization of Phytogenically Synthesized Ag/ZnO Nanomaterials

**Figure 3:**
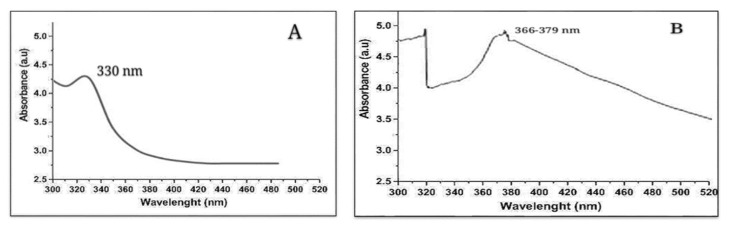

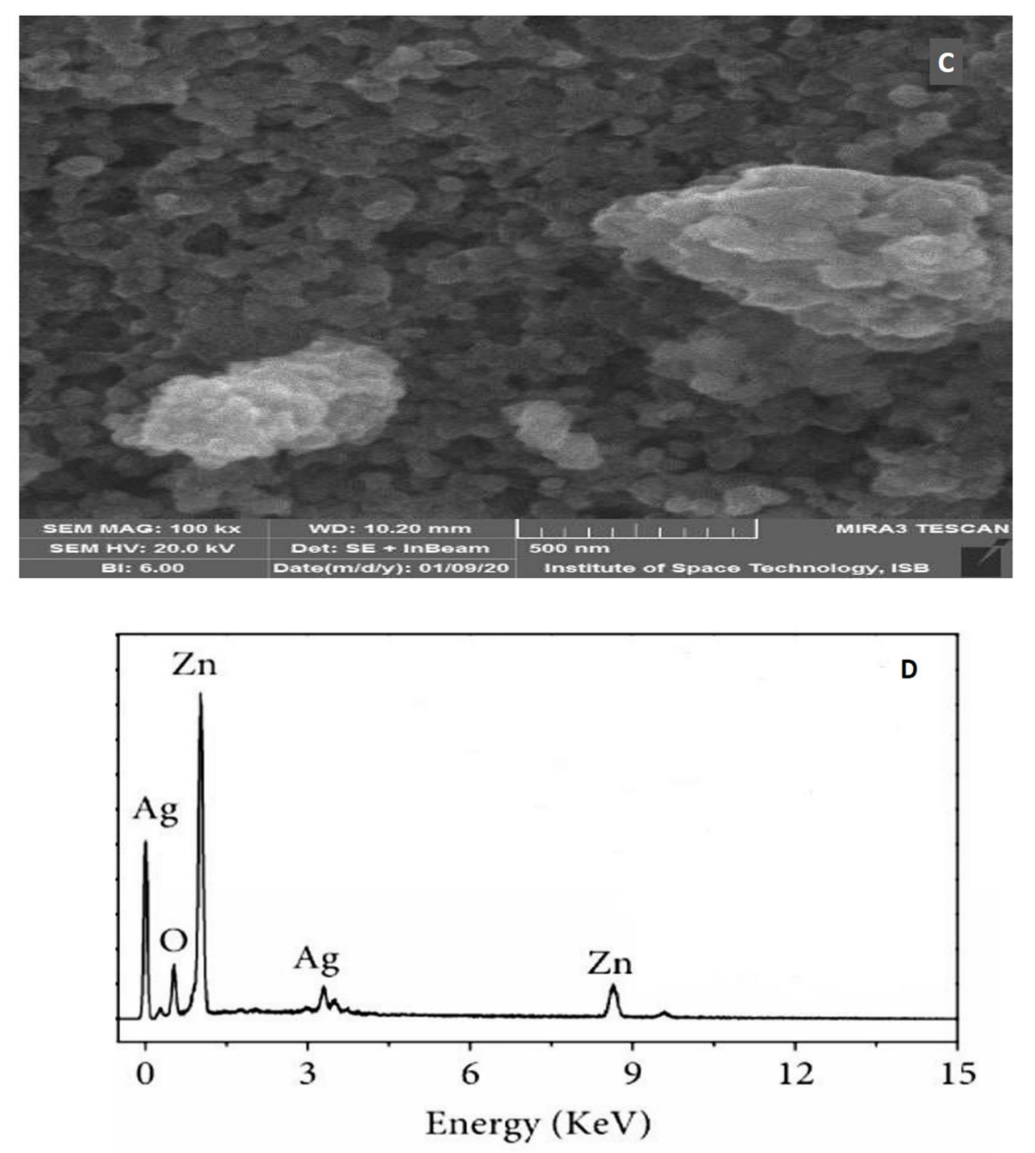

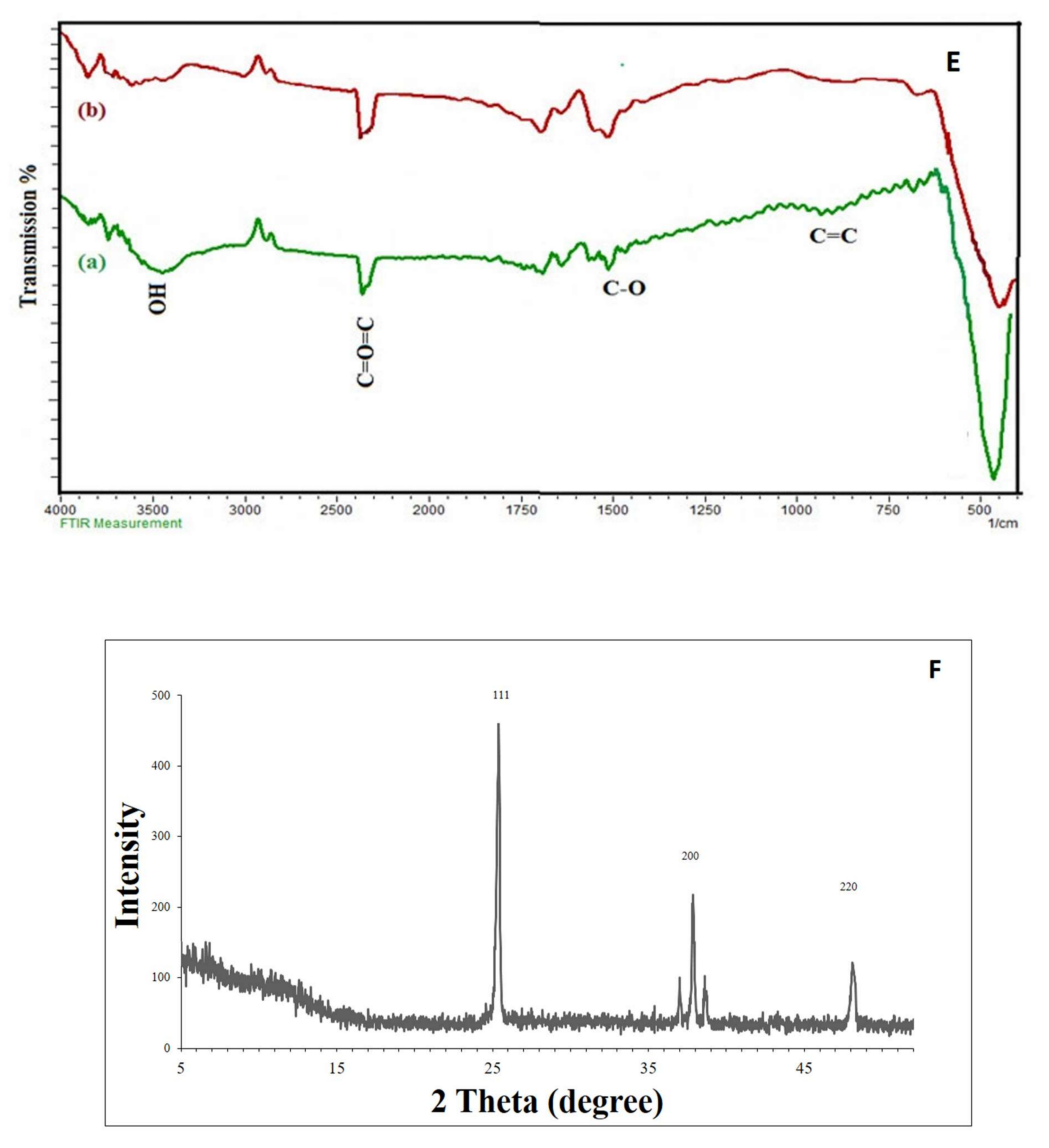
Characterization results of phytogenically synthesized nanomaterials (**A**): UV spectrum of ZnO; (**B**): UV-spectrum of bimetallic Ag/ZnO nanomaterials); (**C**): scanning electron microscopy micrograph of Ag/ZnO nanomaterials; (**D**): EDX spectrum of Ag/ZnO nanomaterials; (**E**): FTIR spectrum of (**a**) ZnO nanomaterials, (**b**) Ag/ZnO nanomaterials; (**F**): XRD spectrum of Ag/ZnO nanomaterials.

Numerous methods have been used for the confirmation of nanomaterials. Among these, UV–visible spectroscopy offers a rudimentary apparatus for verification purposes. In this study, the characterization peaks for ZnO nanomaterials and Ag/ZnO nanomaterials were 330 nm and 366–379 nm, respectively. The structural analysis achieved using SEM revealed the spherical shape of Ag/ZnO nanomaterials with sizes ranging from 46 nm to 66 nm. The presence of Ag and Zn metal was confirmed by the EDX detector. FT-IR measurements were taken (range 450–4000/cm) to recognize and verify ZnO and Ag/ZnO nanomaterials. Absorption bands were detected at 3420 cm^−1^, 1442 cm^−1^, 940 cm^−1^, 470 cm^−1^, 465.06 cm^−1^, and 2352 cm^−1^. These bands correspond to O-H and water, C=O, C=C, Zn-O and Ag-O, C=O=C, and impurities in IR spectra, respectively. The crystalline arrangement of phytogenically synthesized Ag/ZnO nanomaterials was confirmed using XRD spectroscopy (Figure 3A–F).

### 3.2. Role of Phytogenically Synthesized Bimetallic Ag/ZnO Nanomaterials and Nitrogen-based Fertilizers on Biochemical Content of Wheat Varieties

**Figure 4:**
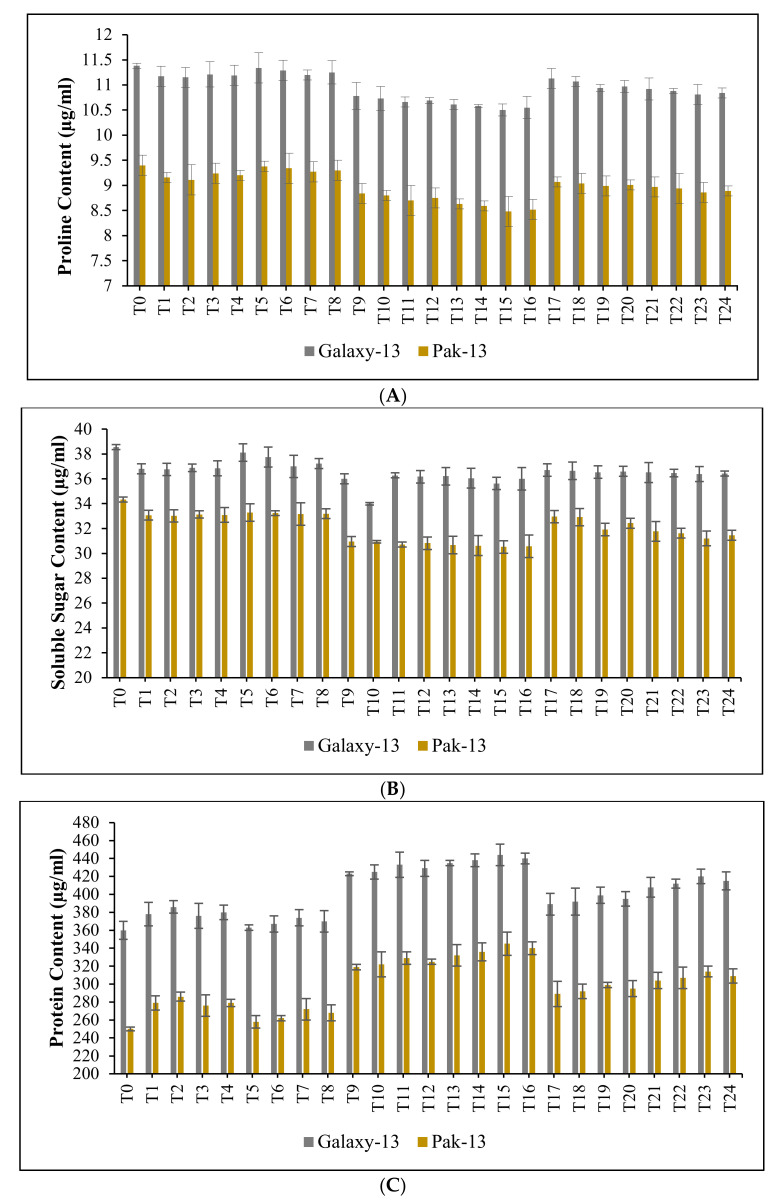
(**A**): effect of phytogenically synthesized bimetallic Ag/ZnO nanomaterials and nitrogen-based fertilizers on proline content of wheat varieties; (**B**): effect of phytogenically synthesized bimetallic Ag/ZnO nanomaterials and nitrogen-based fertilizers on the soluble sugar content of wheat varieties; (**C**): effect of phytogenically synthesized bimetallic Ag/ZnO nanomaterials and nitrogen-based fertilizers on the protein content of wheat varieties.

The biochemical attributes of both wheat varieties were recorded and significant difference (*p* < 0.05) was noted for various concentrations of nitrogenous fertilizers and nanomaterials, including urea (50 mg/L) with NMs, urea (100 mg/L) with NMs, AS (50 mg/L) with NMs, and AS (100 mg/L) with NMs. In comparison with control (T_0_), the following showed significantly different results: T_2_ (urea (100 mg/L)), T_7_ (NMs (60 ppm)), T_11_ (urea (50 mg/L) + NMs (60 ppm)), T_15_ (urea (100 mg/L) + NMs (60 ppm)), T_19_ (AS (50 mg/L) + NMs (60 ppm)), and T_23_ (AS (100 mg/L) + NMs (60 ppm)). Reductions in proline content of 2.04%, 1.59%, 6.53%, 8.04%, 3.94%, and 4.51% were recorded for Galaxy-13 at T_2_, T_7_, T_11_, T_15_, T_19_ and T_23_, respectively. In the case of Pak-13, reductions of proline content recorded at T_2_, T_7_, T_11_, T_15_, T_19,_ and T_23_ were 9.72%, 1.39%, 7.74%, 10.29%, 4.46%, and 5.92%, respectively, compared to control (Figure 4A).

Additionally, soluble sugar content was recorded in both treated and untreated wheat varieties. Sugars are very vigorous osmolytes which assist in defensive mechanisms. According to our results, higher concentrations were associated with higher concentrations of nanomaterials and fertilizers; however, at optimum concentrations of fertilizers and nanomaterials, the results recorded were significant. For Galaxy-13, reductions in soluble sugar content compared to control were noted for T_2_, T_7_, T_11_, T_15_, T_19_ and T_23_ at levels of 4.75%, 4.10%, 6.07%, 7.87%, 5.35%, and 5.79%, respectively; for Pak-13, the corresponding reductions in soluble sugar content were 2.09%, 3.44%, 11.10%, 11.75%, 7.28%, and 9.52%, as illustrated in Figure 4B.

Our research found that the application of optimum concentrations of nitrogen-based fertilizers and biogenic bimetallic Ag/ZnO nanomaterials increased the protein content of bread wheat varieties. Compared to control, protein content increases for T_2_, T_7_, T_11_, T_15_, T_19,_ and T_23_ were 6.97%, 3.81%, 18.41%, 20.90%, 10.28%, and 15.39% in the case of Galaxy-13, while for Pak-13, the corresponding increases for the same treatments were 13.43%, 8.43%, 27.29%, 31.93%, 17.85%, and 22.70%, respectively, as illustrated in Figure 4C.

### 3.3. Role of Phytogenically Synthesized Bimetallic Ag/ZnO Nanomaterials and Nitrogen-based Fertilizers on the Non-Enzymatic Activity of Wheat Varieties

**Figure 5:**
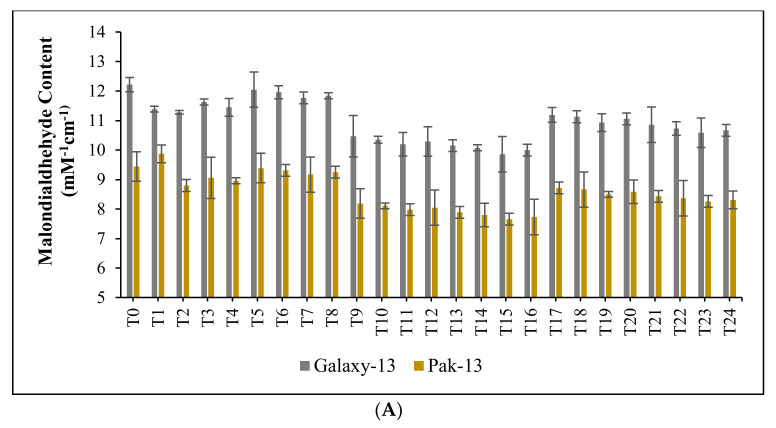

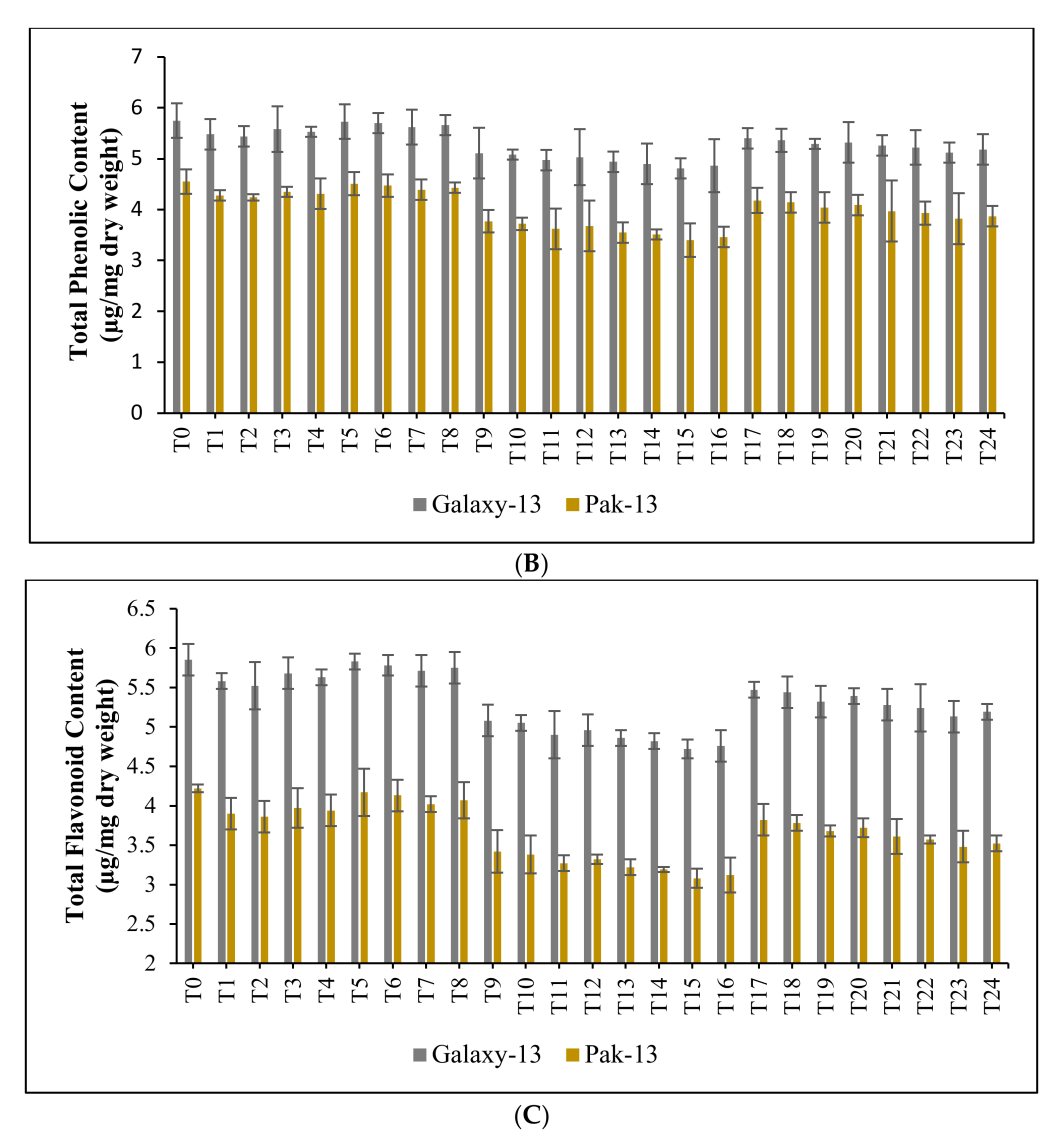
(**A**): effect of phytogenically synthesized bimetallic Ag/ZnO nanomaterials and nitrogen-based fertilizers on malondialdehyde content of wheat varieties; (**B**): effect of phytogenically synthesized bimetallic Ag/ZnO nanomaterials and nitrogen-based fertilizers on total phenolic content of wheat varieties; (**C**): effect of phytogenically synthesized bimetallic Ag/ZnO nanomaterials and nitrogen-based fertilizers on total flavonoid content of wheat varieties.

Compared to control, a significantly decreased concentration of MDA was observed at optimum concentrations of nitrogenous fertilizers and phytogenically synthesized bimetallic Ag/ZnO nanomaterials. In the case of Galaxy-13, MDA levels decreased by 8%, 3.75%, 18.02%, 21.38%, 11.5%, and 14.29%, at T_2_, T_7_, T_11_, T_15_, T_19,_ and T_23_, respectively; however, for Pak-13 the corresponding decreases were 7.02%, 2.90%, 16.76%, 20.82%, 10.48%, and 13.33%, as illustrated in Figure 5A. 

Secondary metabolites such as phenolic and flavonoid contents were also identified in both wheat varieties. Statistically significant decreases in both phenolic and flavonoid content were recorded for various concentrations of fertilizers and nanomaterials in comparison with control. In the case of Galaxy-13, total phenolic content decreased by 5.54%, 2.29%, 14.55%, 17.80%, 8.33%, and 11.61%, while total flavonoid content decreased by 5.80%, 2.44%, 17.67%, 21.38%, 9.40% and 13.12%, at T_2_, T_7_, T_11_, T_15_, T_19_ and T_23,_ respectively. In the case of Pak-13, total phenolic content decreased by 7.05%, 3.58%, 23.99%, 28.93%, 11.87%, and 17.44%, while total flavonoid content decreased by 8.91%, 4.85%, 25.37%, 31.23%, 13.67%, and 19.22%, at T_2_, T_7_, T_11_, T_15_, T_19,_ and T_23,_ respectively (Figure 5B,C).

### 3.4. Role of Phytogenically Synthesized Bimetallic Ag/ZnO Nanomaterials and Nitrogen-Based Fertilizers on Antioxidant Enzymes Activity of Wheat Varieties

**Figure 6:**
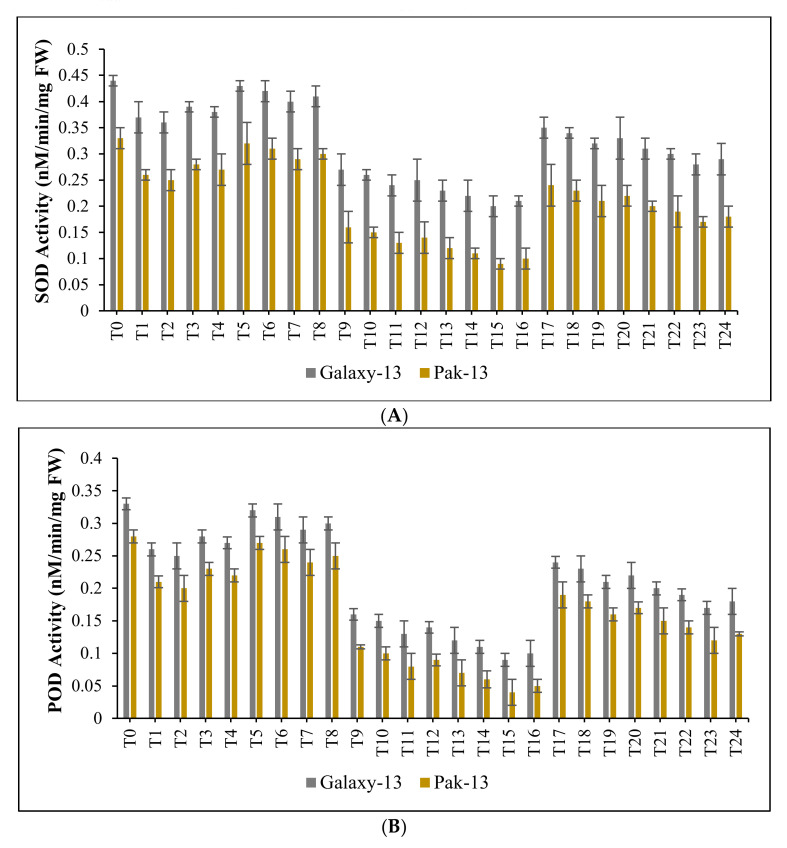

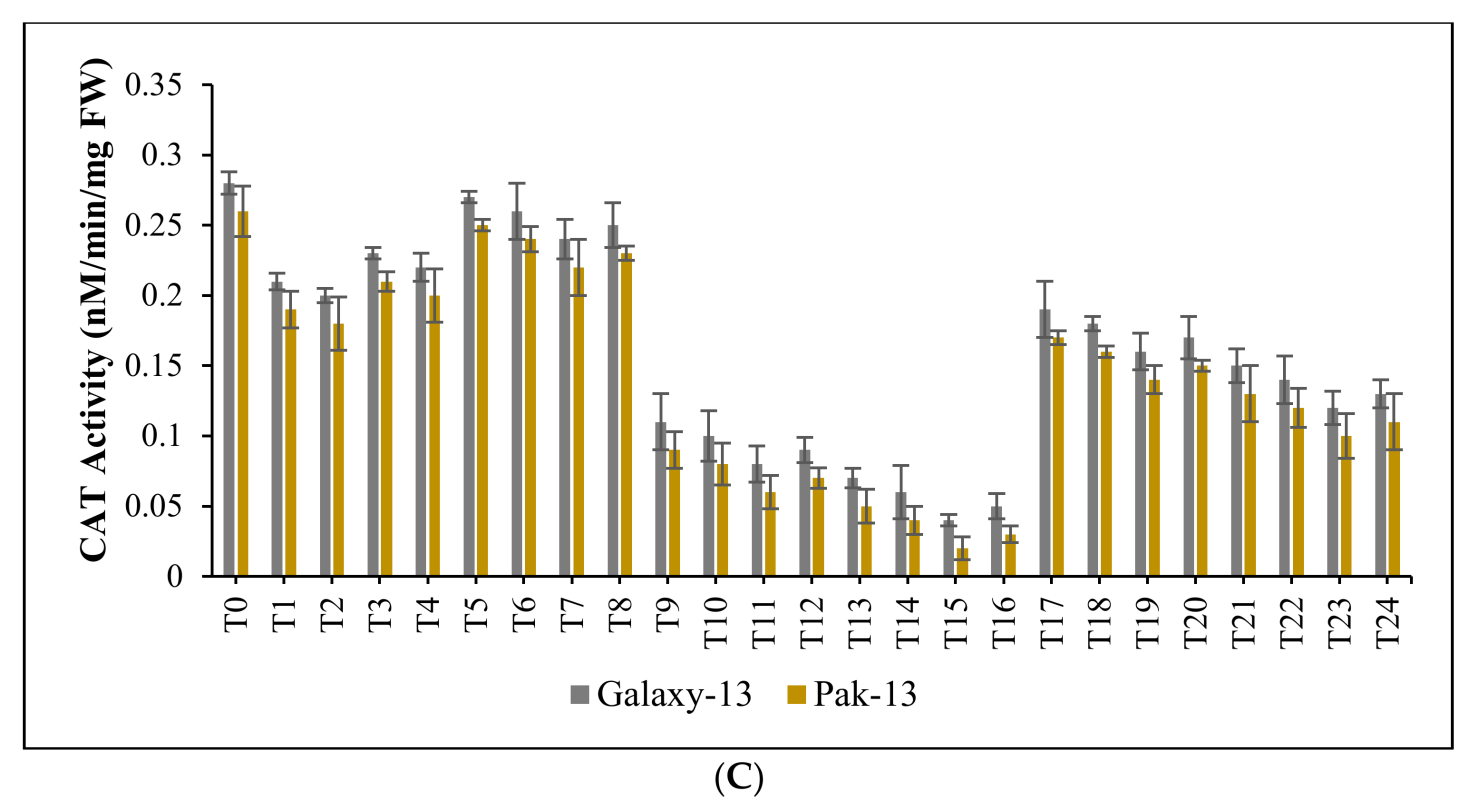
(**A**): effect of phytogenically synthesized bimetallic Ag/ZnO nanomaterials and nitrogen-based fertilizers on SOD activity of wheat varieties; (**B**): effect of phytogenically synthesized bimetallic Ag/ZnO nanomaterials and nitrogen-based fertilizers on POD activity of wheat varieties; (**C**): effect of phytogenically synthesized bimetallic Ag/ZnO nanomaterials and nitrogen-based fertilizers on CAT activity of wheat varieties.

In this study, the responses of principal antioxidant enzymes such as SOD, POD, and CAT to certain concentrations of Ag/ZnO nanomaterials and nitrogenous fertilizers were also observed for both wheat varieties. Compared to control, significant decreases in levels of SOD, POD and CAT were observed in Galaxy-13 at T_2_, T_7_, T_11_, T_15_, T_19_ and T_23_. Specifically, for SOD, the reductions were 20%, 9.52%, 58.82%, 75%, 31.58%, and 44.44%; for POD, the reductions were 27.59%, 12.90%, 86.96%, 114.29%, 44.44% and 64%; while, for CAT, the reductions were 33.33%, 15.39%, 111.11%, 150%, 54.55% and 80%, respectively. In the case of Pak-13, significant decreases in SOD, POD and CAT were also observed at T_2_, T_7_, T_11_, T_15_, T_19,_ and T_23_. Specifically, for SOD, the reductions were 28.58%, 13.52%, 88.82%, 115%, 49.58%, and 69.44%; for POD, the reductions were 36.59%, 16.90%, 115.96%, 158.29%, 57.44%, and 84%; while for CAT, the reductions were 36.36%, 16.67%, 125%, 171.43%, 60%, and 88.89%, respectively (Figure 6A–C).

### 3.5. Role of Phytogenically Synthesized Bimetallic Ag/ZnO Nanomaterials and Nitrogen-based Fertilizers on Yield Attributes of Wheat Varieties

**Figure 7:**
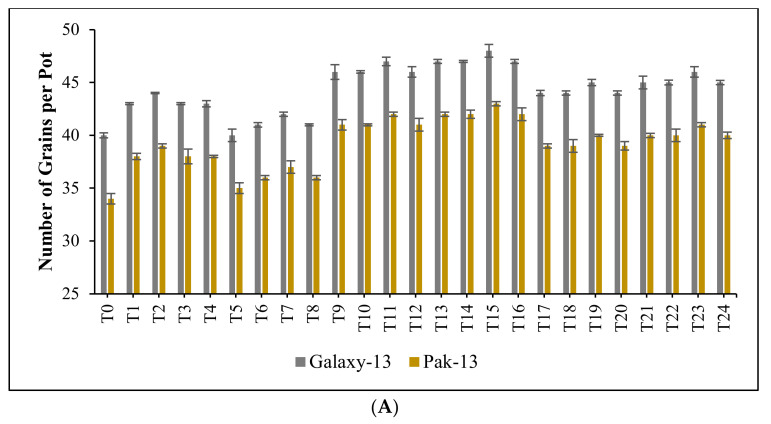

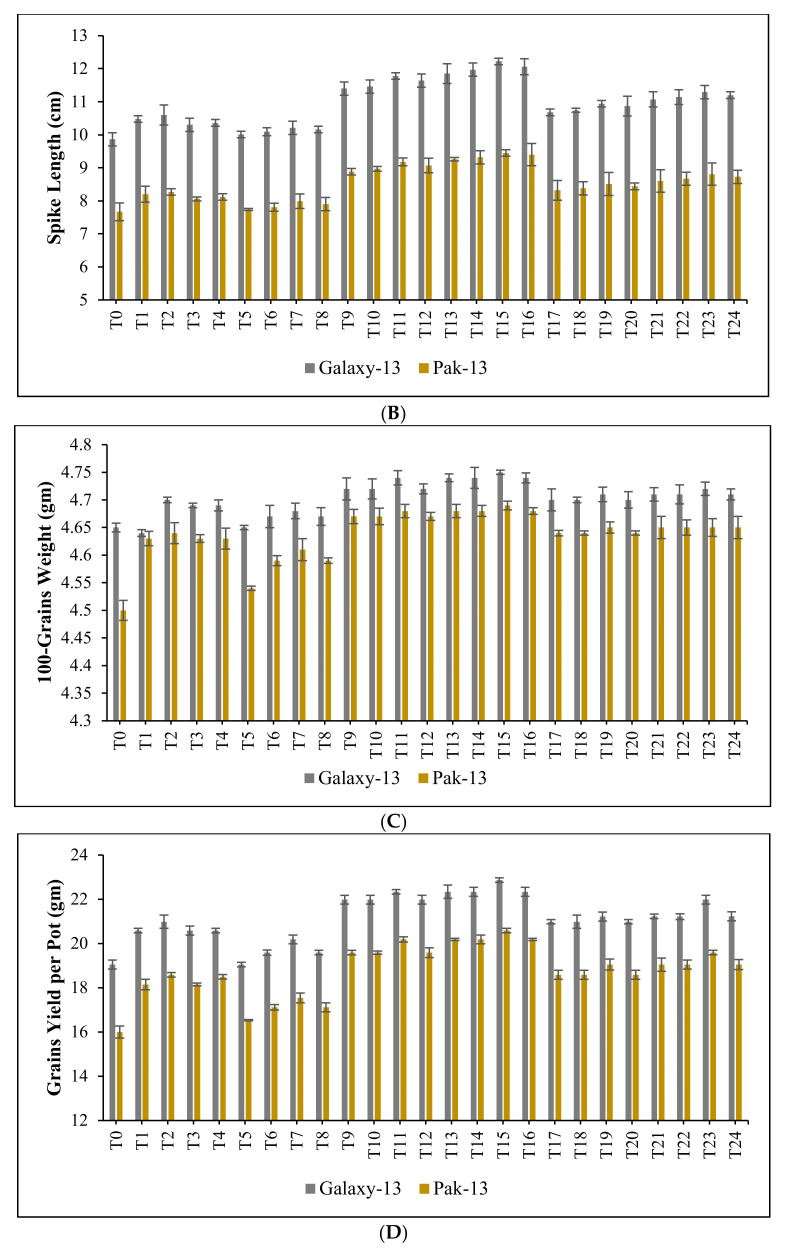

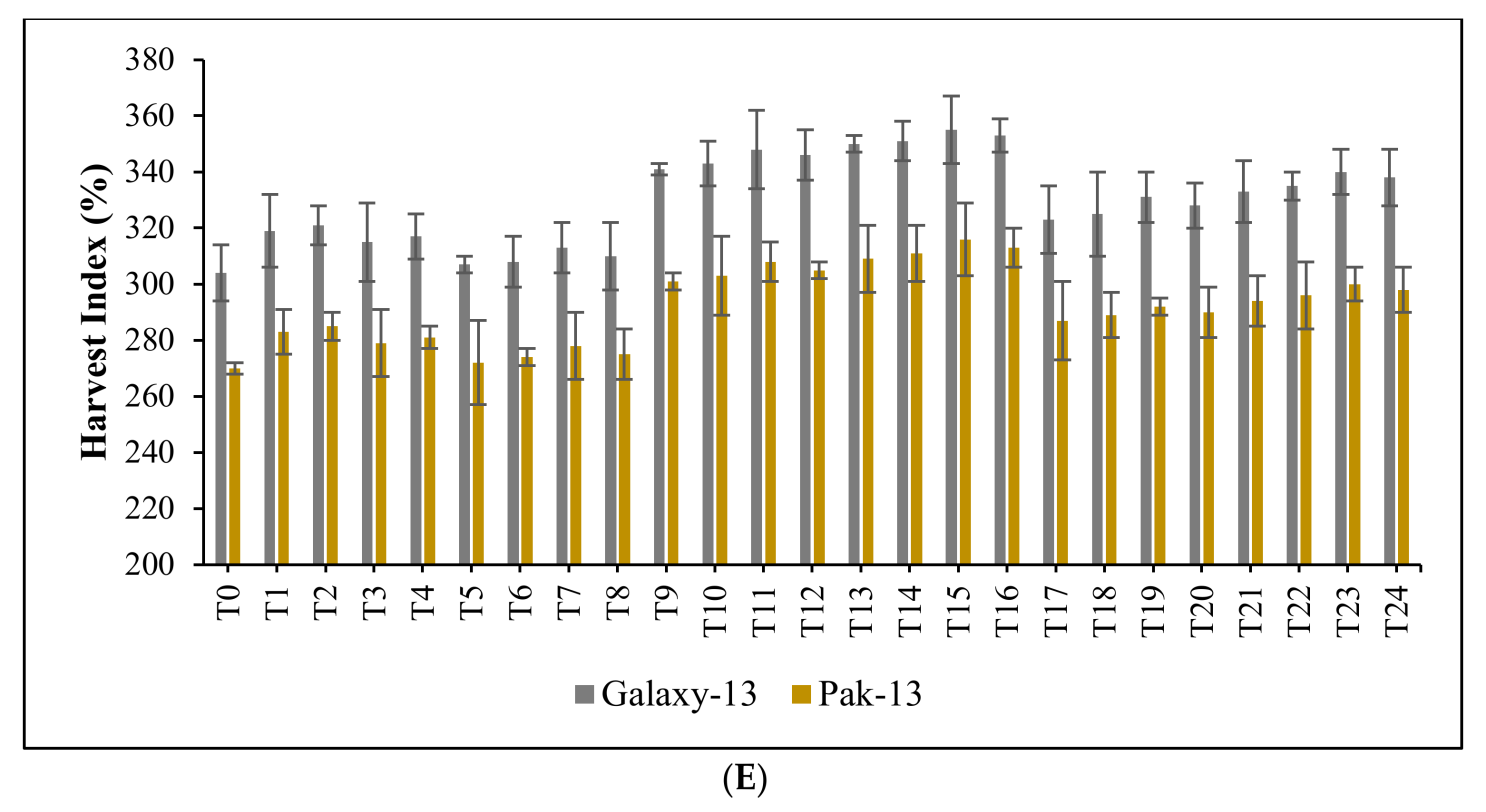
(**A**): effect of phytogenically synthesized bimetallic Ag/ZnO nanomaterials and nitrogen-based fertilizers on the number of grains per pot of both wheat varieties; (**B**): effect of phytogenically synthesized bimetallic Ag/ZnO nanomaterials and nitrogen-based fertilizers on spike length of both wheat varieties; (**C**):effect of phytogenically synthesized bimetallic Ag/ZnO nanomaterials and nitrogen-based fertilizers on 100-grain weight of both wheat varieties; (**D**): effect of phytogenically synthesized bimetallic Ag/ZnO nanomaterials and nitrogen-based fertilizers on grain yield per pot of both wheat varieties; (**E**): effect of phytogenically synthesized bimetallic Ag/ZnO nanomaterials and nitrogen-based fertilizers on harvest index of both wheat varieties.

The yield attributes of wheat varieties Galaxy-13 and Pak-13 at maturity were investigated and it was observed that optimum concentrations of bimetallic Ag/ZnO nanomaterial and nitrogen-based fertilizers resulted in increased yield parameters in both varieties compared to control. At T_2_, T_7_, T_11_, T_15_, T_19_ and T_21_, significantly increased numbers of grains per pot were observed. For Galaxy-13, the increases were 9.52%, 4.88%, 16.09%, 18.18%, 11.77%, and 13.95%, respectively. For Pak-13, the corresponding increases were 13.70%, 8.45%, 21.05%, 23.78%, 16.22%, and 18.67% in Pak-13. Similarly, at T_2_, T_7_, T_11_, T_15_, T_19_ and T_23_, increases in spike length were recorded of 7.84%, 3.49%, 17.74%, 21.38%, 10.39%, and 13.52% for Galaxy-13, with corresponding increases of 7.53%, 4.09%, 17.92%, 20.79%, 10.38%, and 13.84% for Pak-13 (Figure 7A,B).

Increases in 100-grain weights and grain yields per pot were also produced by foliar application of nanomaterials and fertilizers. In the case of Galaxy-13, at T_2_, T_7_, T_11_, T_15_, T_19,_ and T_23,_ increases in 100-grain weight were 7.84%, 3.49%, 17.74%, 21.38%, 10.39% and 13.92%; for Pak-13, the corresponding increases were 3.06%, 2.42%, 3.92%, 4.13%, 3.28%, and 3.29%. Significant increases in grain yields were also recorded. For Galaxy-13, at T_2_, T_7_, T_11_, T_15_, T_19_ and T_23_, these increases were 9.69%, 5.66%, 15.90%, 18.23%, 14.32% and 14.28%, respectively, while for Pak-13, the corresponding increases were 14.98%, 9.18%, 23.16%, 25.09%, 17.40%, and 20.17%, respectively, when compared to control (Figure 7C,D).

In the present study, the harvest index of both wheat varieties at maturity was also determined. Here, again, significantly increased values were recorded after the application of bimetallic Ag/ZnO nanomaterials and nitrogen-based fertilizers. In the case of Galaxy-13, at T_2_, T_7_, T_11_, T_15_, T_19_ and T_23_ the index values increased by 5.44%, 2.92%, 13.50%, 15.48%, 8.50%, and 11.18%; for Pak-13, the corresponding increases in the harvest index were 5.41% 2.92%, 13.15%, 15.70%, 7.83%, and 10.53%, compared to control (Figure 7E).

## 4. Discussion

Because of increasing world population, rising demand for food requires the use of fertilizers on a large scale. Because of resource limitation and declining fertilizer efficiency, costs to farmers have risen rapidly [41]. After carbon, hydrogen and oxygen, nitrogen is the basic nutrient essential for growth, phytohormones, the photosynthetic process, proteomic modification and development in plants [42]. Excessive as well as inefficient usage of nitrogen-based fertilizers results in increased crop production costs as well as environmental contamination. Looking ahead, considering the need for sustainable production of food and eco-friendly paybacks worldwide, there is a critical requirement to up-regulate the efficiency of nutrient use in the agrarian system [43]. In a similar way to macronutrients, micronutrients are also very important for plants and their functions include the development of root and respiratory cells, metabolism linked with chlorophyll formation, photosynthetic activity, and metabolic enzyme efficiency [44].

Nanotechnology is emerging as the most beneficial technology for future food security, with the potential to improve fertilizers production using modified chemical composition, improving the efficiency of nutrient use, boosting crop yields and at the same time reducing ecological impact [45]. This technology involves the production and strategic application of metal-derived nanomaterials with a dimension of less than 0.1 micrometers. Exploration of the field emerged around the turn of the 21st century, when nanomaterials first revealed their potential agricultural uses based on their distinctive physicochemical characteristics [46]. In recent years, there has also been an increase in the use of metal-based nanoparticles such as bimetallic nanomaterials in daily life. Phytogenically prepared silver/zinc oxide-based nanomaterials are already used in a wide range of applications in the fields of medicine, industry, and agriculture [47,48].

**Figure 8:**
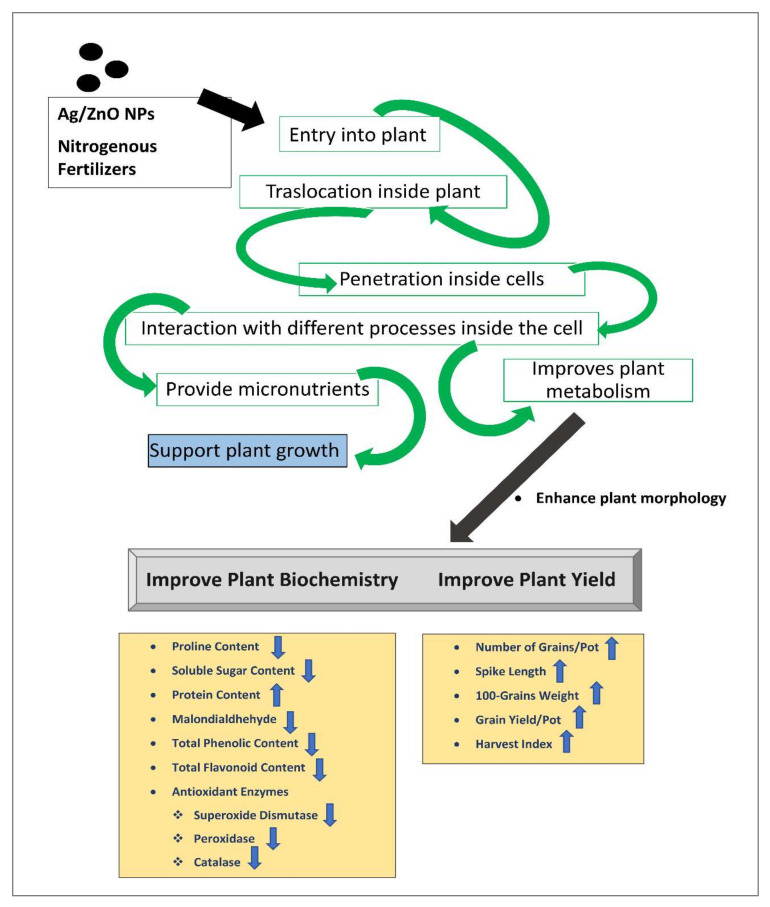
Overall effects of phytogenically synthesized bimetallic Ag/ZnO nanomaterials and nitrogens-based fertilizers on wheat plants.

Bimetallic-based nanoparticles such as silver/zinc oxide, have been used beneficially for their anti-cancer, antioxidant, antidiabetic, bio-molecular detection and secondary metabolite qualities. However, the use of bimetallic nanomaterials such as Ag/ZnO in the pursuit of plant growth and development, food production, nutrient use efficiency, and food security has not been so widespread as the use of monometallic nanomaterials such as gold, silver, zinc, copper, titanium, and iron for such purposes [49,50]. These metal-based nanomaterials increase the growth and yield of crops and perform a valuable role against biotic and abiotic stresses. The application of plant-derived Ag/ZnO nanomaterials as a means of promoting growth is a recent innovation in the agricultural field of nano-biotechnology [51]. The use of bimetallic Ag/ZnO nanomaterials as nano-fertilizers, with their synergistic effects, is more beneficial compared to monometallic nanomaterials, on account of the higher surface-area-to-volume ratio of the former. This property allows slow and controlled release, hence promoting effective nutrient uptake by the crops. Therefore, this technique can be seen as a foundation for new and sustainable nutrient distribution systems [52]. An illustration of the overall effects of nitrogenous fertilizers and Ag/ZnO on wheat varieties has been incorporated into this study (Figure 8).

Plant-derived nanomaterials have many advantages compared to chemically synthesized nanomaterials in terms of cost and toxicity. In this research, Ag/ZnO nanomaterials were synthesized using leaf extract of *Moringa oleifera* L. Various phytochemicals such as aldehyde, amine, carboxylic acids, flavonoids, ketones, terpenoids, and quinines present in leaf extract perform a key role as reducing and capping agents in the production of plant-derived Ag/ZnO nanomaterials [15].

### 4.1. Characterization of Phytogenically Synthesized Ag/ZnO Nanomaterials

Previous studies have shown that the UV-visible spectrum for metal-metal oxide nanomaterials ranges from 340 to 400 nm [53]. Similar results related to the morphology of nanomaterials have been described and explained by other researchers [54]. The presence of silver and zinc metal was also verified by other researchers through EDX [55]. The FTIR spectrum indicates the presence of various compounds such as hydroxyl, carboxyl, carbonyl, ketones, and so on, which have also been reported in the literature for their role as reducing and capping agents for the plant-derived synthesis of Ag/ZnO nanomaterials [56]. Our XRD spectrum results, indicating crystalline structure, are also in line with previous research [57].

### 4.2. Role of Phytogenically Synthesized Bimetallic Ag/ZnO Nanomaterials and Nitrogenous-Based Fertilizers on the Biochemistry of Wheat Varieties

In the present study, a decrease in proline content and soluble sugar content was noted in both wheat varieties when treated with plant-derived Ag/ZnO nanomaterials along with nitrogenous fertilizers. At the same time, the increase in wheat production suggests a successful defense against reactive oxygen species mediating oxidative stress due to higher concentrations of nanomaterials. In this regard, green synthesized Ag/ZnO (60 ppm) + urea (100 mg/L) significantly decreases the production of proline and soluble sugar content by reducing ROS activity. Our results are in line with previous literature citing nanomaterial-arbitrated reductions in proline and sugar accumulation in various plants [58].

A significant increase in protein content was observed in both Galaxy-13 and Pak-13 due to the foliar application of nitrogen-based fertilizers and plant-derived bimetallic Ag/ZnO nanomaterials. Similarly, the authors of [59] noted increased protein production in crop plants due to the application of green synthesized nanomaterials, while the application of fertilizers also enhanced protein content in crops [60].

### 4.3. Role of Phytogenically Synthesized Bimetallic Ag/ZnO Nanomaterials and Nitrogen-Based Fertilizers on Secondary Metabolites of Wheat Varieties

The accumulation of MDA occurs when lipids of plasma membrane break down. However, stress induces lipid peroxidation. In this study, a higher concentration of nanomaterials along with nitrogen-based fertilizers resulted in increased MDA production in both wheat varieties representing plasma membrane damage. MDA levels in wheat plants decreased significantly with foliar applications of 60 ppm concentration Ag/ZnO nanomaterials with nitrogen-based fertilizers (urea and ammonium sulphate). This optimum concentration of plant-derived nanomaterials led to lower levels of lipid peroxidation. Our findings are similar to those of Landa [61] who noted decreased MDA production in plants with green synthesized metal-based nanoparticles.

The present study found reductions in total phenolic and total flavonoid content in both wheat varieties when exposed to 60 ppm concentration of plant-derived Ag/ZnO nanomaterials with 50 mg/L and 100 mg/L of urea and ammonium sulphate. Similarly, athe authors of [62,63] also demonstrated the accumulation of non-enzymatic antioxidants (TPC and TFC) due to various nanomaterials in plants. The production and transfer of secondary metabolites in wheat plants are regulated by silver and zinc oxide-based nanomaterials which enhance the growth of wheat plants [64].

### 4.4. Role of Phytogenically Synthesized Bimetallic Ag/ZnO Nanomaterials and Nitrogen-based Fertilizers on Antioxidant Enzymes of Wheat Varieties

Plants have various enzymatic and non-enzymatic outlines for reactive oxygen species incorporating compounds. APX, CAT, DHAR, GR, GST, SOD and POD are examples of enzymatic outlines, while non-enzymatic mixtures include ascorbate, GSH, etc. [65]. The synthesis and accumulation of enzymatic antioxidants are associated with various kinds of stresses including high concentrations of minerals and metals upsetting the physiology of plants. In this study, increased synthesis of SOD, POD, and CAT were observed when plants were treated with higher concentrations of nanomaterials and fertilizers, alleviating unwanted marks of stressful conditions in Galaxy-13 and Pak-13. Because of the application of plant-derived nanomaterials (60 ppm) with urea and ammonium sulphate, a significant decrease in enzymatic antioxidants (SOD, POD, and CAT) was observed in both wheat varieties. Farooq et al., [66] reported that the activity of all the antioxidative enzymes was prolonged in sum at all stages of growth in plants. Alabdallah et al., [67] reported decrease in the production of SOD, POD, and CAT on crop plants by application of phytogenically prepared metal-based nanomaterials.

### 4.5. Role of Phytogenically Synthesized Bimetallic Ag/ZnO Nanomaterials and Nitrogen-based Fertilizers on Yield Attributes of Wheat Varieties

Yield attributes such as the number of grains per pot, spike length, 100-grain weight, grain yield per pot, and harvest index are considered to be important parameters when studying the success of wheat plants. These yield parameters of Galaxy-13 and Pak-13 were examined after plant-derived treatment with Ag/ZnO nanomaterials and fertilizers (urea and ammonium sulphate). All the plants showed significantly higher yields and productivity when treated with 60 ppm Ag/ZnO nanomaterials with nitrogenous fertilizers. This increase in yield parameters illustrates the positive effects of phytogenically synthesized Ag/ZnO nanomaterials and fertilizers on both wheat varieties. These findings are similar to the results of [68] which describe increased yield attributes in plants treated with green synthesized metal-based nanomaterials. Other scientists [69,70] have also revealed the role of urea and ammonium sulphate in improving yields of various plants.

## 5. Conclusion and Future Recommendations

Wheat is Pakistan’s most important food crop, exceeding all other crops in terms of land usage and productivity. Reduced use of nitrogenous fertilizers results in lower yields leading to scarcity and hunger. The use of nanomaterials as fertilizers can boost crop yieds without any harmful effects on soil. The biogenic approach is more effective and less expensive than physical or chemical approaches to the synthesis of nanomaterials. It paves the way for a forthcoming era of bio-nanotechnology. Ag- and ZnO-based NPs have been used in farming for the improvement of crops by promoting germination, raising the seedling vigor index, and improving the growth of wheat seeds. Under such conditions. When seeds of wheat plants germinate in the soil, their growth rate increases, and production rates rise by 20–25 percent. The main objective of this study was to investigate the effects of various concentrations of phytogenically synthesized Ag/ZnO nanomaterials and nitrogen-based fertilizers on the biochemistry and yield of two wheat varieties (Galaxy-13 and Pak-13). Leaf extract of *Moringa oleifera* L. was the major reducing and capping agent for the synthesis of Ag/ZnO nanomaterials and was characterized using UV–visible spectroscopy, SEM, FTIR, EDX, and XRD techniques. It was observed that Ag/ZnO nanomaterials (60 ppm) with various concentrations of urea and ammonium sulphate produced a significantly positive effect by improving the biochemical and yield attributes of both varieties. Therefore, we conclude that phytogenically prepared Ag/ZnO nanomaterials have the potential to improve biochemistry, thereby enhancing yield. Further comprehensive studies are needed into agro-morphological changes in wheat varieties. Studies on biochemistry and yields should be undertaken on a larger scale in future. Furthermore, proteome and genome analysis could be carried out on wheat varieties treated with these nanomaterials and fertilizers for a detailed demonstration of their role at the molecular level.

## Figures and Tables

**Table 1:** Experimental layout of treatments (AS: ammonium sulphate, NMs: nanomaterials).

Treatments	Description	Treatments	Description
** T1 **	Urea 50 mg/L	**T13**	Urea 100 mg/L + NMs 20 ppm
** T2 **	Urea 100 mg/L	**T14**	Urea 100 mg/L + NMs 40 ppm
** T3 **	AS 50 mg/L	**T15**	Urea 100 mg/L + NMs 60 ppm
** T4 **	AS 100 mg/L	**T16**	Urea 100 mg/L + NMs 80 ppm
** T5 **	NMs 20 ppm	**T17**	AS 50 mg/L + NMs 20 ppm
** T6 **	NMs 40 ppm	**T18**	AS 50 mg/L + NMs 40 ppm
** T7 **	NMs 60 ppm	**T19**	AS 50 mg/L + NMs 60 ppm
** T8 **	NMs 80 ppm	**T20**	AS 50 mg/L + NMs 80 ppm
** T9 **	Urea 50 mg/L + NMs 20 ppm	**T21**	AS 100 mg/L + NMs 20 ppm
** T10 **	Urea 50 mg/L + NMs 40 ppm	**T22**	AS 100 mg/L + NMs 40 ppm
** T11 **	Urea 50 mg/L + NMs 60 ppm	**T23**	AS 100 mg/L + NMs 60 ppm
** T12 **	Urea 50 mg/L + NMs 80 ppm	**T24**	AS 100 mg/L + NMs 80 ppm

## Data Availability

All data collected or analyzed during this research are included ino this article.

## References

[1] Lafiandra D., Sestili F., Sissons M., Kiszonas A., Morris C.F. (2022). Increasing the Versatility of Durum Wheat through Modifications of Protein and Starch Composition and Grain Hardness. Foods.

[2] Ullah I., Muhammad D., Mussarat M. (2022). Effect of Various Nitrogen Sources at Various Sulfur Levels on Maize–Wheat Yield and N/S Uptake under Different Climatic Conditions. J. Plant Growth Regul..

[3] Chen C., Zhou S., Afshar R.K., Franck W., Zhou Y. (2022). Durum wheat yield and protein influenced by nitrogen management and cropping rotation. J. Plant Nutr..

[4] Wang X. (2022). Managing Land Carrying Capacity: Key to Achieving Sustainable Production Systems for Food Security. Land.

[5] Gessesew W.S., Elias E., Gebresamuel G., Tefera W. (2022). Soil type and fertilizer rate affect wheat (*Triticum aestivum* L.) yield, quality and nutrient use efficiency in Ayiba, northern Ethiopia. PeerJ.

[6] Barrett C.B. (2021). Overcoming global food security challenges through science and solidarity. Am. J. Agric. Econ..

[7] Singh D., Arya S., Gupta B., Kaushik D., Arya V.S., Kumar U., Singh K. (2021). Applications of nanotechnology in forest management. J. Nanosci. Nanotechnol..

[8] Karuppannan S.K., Dowlath M.J.H., Ramachandran S., Rajadesingu S., Arunachalam K.D. (2022). Interaction of nanomaterials with microbes. Nano-Bioremediation: Fundamentals and Applications.

[9] Fatima F., Hashim A., Anees S. (2021). Efficacy of nanoparticles as nanofertilizer production: A review. Environ. Sci. Pollut. Res..

[10] Basit F., Asghar S., Ahmed T., Ijaz U., Noman M., Hu J., Liang X., Guan Y. (2022). Facile synthesis of nanomaterials as nanofertilizers: A novel way for sustainable crop production. Environ. Sci. Pollut. Res..

[11] Gondal A.H., Tayyiba L. (2022). Prospects of Using Nanotechnology in Agricultural Growth, Environment and Industrial Food Products. Rev. Agric. Sci..

[12] Iqbal M.A. (2019). Nano-fertilizers for sustainable crop production under changing climate: A global perspective. Sustain. Crop Prod..

[13] Ndaba B., Roopnarain A., Rama H., Maaza M. (2022). Biosynthesized metallic nanoparticles as fertilizers: An emerging precision agriculture strategy. J. Integr. Agric..

[14] Sarraf M., Vishwakarma K., Kumar V., Arif N., Das S., Johnson R., Janeeshma E., Puthur J.T., Aliniaeifard S., Chauhan D.K. (2022). Metal/metalloid-based nanomaterials for plant abiotic stress tolerance: An overview of the mechanisms. Plants.

[15] Mazhar T., Shrivastava V., Tomar R.S. (2017). Green synthesis of bimetallic nanoparticles and its applications: A review. J. Pharm. Sci. Res..

[16] Sharma G., Kumar A., Sharma S., Naushad M., Dwivedi R.P., ALOthman Z.A., Mola G.T. (2019). Novel development of nanoparticles to bimetallic nanoparticles and their composites: A review. J. King Saud Univ. Sci..

[17] Sharma R., Tripathi A. (2022). Green synthesis of nanoparticles and its key applications in various sectors. Mater. Today Proc..

[18] Doolotkeldieva T., Bobusheva S., Zhasnakunov Z., Satybaldiev A. (2022). Biological Activity of Ag and Cu Monometallic Nanoparticles and Ag-Cu Bimetallic Nanocomposites against Plant Pathogens and Seeds. J. Nanomater..

[19] Saleem H., Zaidi S.J. (2020). Recent developments in the application of nanomaterials in agroecosystems. Nanomaterials.

[20] Fen L.B., Rashid A.H.A., Nordin N.I., Hossain M.M., Uddin S.M.K., Johan M.R., Thangadurai D. (2022). Applications of Nanomaterials in Agriculture and Their Safety Aspect. Biogenic Nanomaterials.

[21] Khalaki M.A., Moameri M., Ghorbani A., Alagoz S.M., Dolatabadi N., Lajayer B.A., van Hullebusch E.D. (2022). Effects, uptake and translocation of Ag-based nanoparticles in plants. Toxicity of Nanoparticles in Plants.

[22] Hassanisaadi M., Barani M., Rahdar A., Heidary M., Thysiadou A., Kyzas G.Z. (2022). Role of agrochemical-based nanomaterials in plants: Biotic and abiotic stress with germination improvement of seeds. Plant Growth Regul..

[23] Al Jabri H., Saleem M.H., Rizwan M., Hussain I., Usman K., Alsafran M. (2022). Zinc Oxide Nanoparticles and Their Biosynthesis: Overview. Life.

[24] Sarkar M.R., Rashid M.H.O., Rahman A., Kafi M.A., Hosen M.I., Rahman M.S., Khan M.N. (2022). Recent advances in nanomaterials based sustainable agriculture: An overview. Environ. Nanotechnol. Monit. Manag..

[25] Bhardwaj A.K., Arya G., Kumar R., Hamed L., Pirasteh-Anosheh H., Jasrotia P., Lal Kashyap P., Singh G.P. (2022). Switching to nanonutrients for sustaining agroecosystems and environment: The challenges and benefits in moving up from ionic to particle feeding. J. Nanobiotechnol..

[26] Bayat M., Zargar M., Murtazova KM S., Nakhaev M.R., Shkurkin S.I. (2022). Ameliorating Seed Germination and Seedling Growth of Nano-Primed Wheat and Flax Seeds Using Seven Biogenic Metal-Based Nanoparticles. Agronomy.

[27] Singhal J., Verma S., Kumar S. (2022). The physio-chemical properties and applications of 2D nanomaterials in agricultural and environmental sustainability. Sci. Total Environ..

[28] Sorbiun M., Shayegan Mehr E., Ramazani A., Taghavi Fardood S. (2018). Biosynthesis of Ag, ZnO and bimetallic Ag/ZnO alloy nanoparticles by aqueous extract of oak fruit hull (Jaft) and investigation of photocatalytic activity of ZnO and bimetallic Ag/ZnO for degradation of basic violet 3 dye. J. Mater. Sci. Mater. Electron..

[29] Hosny M., Fawzy M., Eltaweil A.S. (2022). Green synthesis of bimetallic Ag/ZnO@ Biohar nanocomposite for photocatalytic degradation of tetracycline, antibacterial and antioxidant activities. Sci. Rep..

[30] Bates L.S., Waldren R.P., Teare I.D. (1973). Rapid determination of free proline for water-stress studies. Plant Soil.

[31] Dubois M., Gilles K.A., Hamilton J.K., Rebers P.A., Smith F.A.J.N. (1951). A colorimetric method for the determination of sugars. Nature.

[32] Classics Lowry O., Rosebrough N., Farr A., Randall R. (1951). Protein measurement with the Folin phenol reagent. J. Biol. Chem..

[33] Bailly C., Benamar A., Corbineau F., Côme D. (1996). Changes in malondialdehyde content and in superoxide dismutase, catalase and glutathione reductase activities in sunflower seeds as related to deterioration during accelerated aging. Physiol. Plant..

[34] Giri L., Dhyani P., Rawat S., Bhatt I.D., Nandi S.K., Rawal R.S., Pande V. (2012). In vitro production of phenolic compounds and antioxidant activity in callus suspension cultures of *Habenaria edgeworthii*: A rare Himalayan medicinal orchid. Ind. Crops Prod..

[35] Velioglu Y., Mazza G., Gao L., Oomah B.D. (1998). Antioxidant activity and total phenolics in selected fruits, vegetables, and grain products. J. Agric. Food Chem..

[36] Chang C.C., Yang M.H., Wen H.M., Chern J.C. (2002). Estimation of total flavonoid content in propolis by two complementary colorimetric methods. J. Food Drug Anal..

[37] Nayyar H., Gupta D. (2006). Differential sensitivity of C3 and C4 plants to water deficit stress: Association with oxidative stress and antioxidants. Environ. Exp. Bot..

[38] Aucique Pérez C.E. (2016). Wheat Resistance to Blast Using a Non-Host Selective Toxin and Host Metabolic Reprogramming through a Successful Infection by *Pyricularia oryzae*. Ph.D. Thesis.

[39] Lagrimini L.M. (1991). Wound-induced deposition of polyphenols in transgenic plants overexpressing peroxidase. Plant Physiol..

[40] Aebi H. (1984). Catalase in vitro. Methods in Enzymology.

[41] Magrach A., Sanz M.J. (2020). Environmental and social consequences of the increase in the demand for ‘superfoods’ world-wide. People Nat..

[42] Anas M., Liao F., Verma K.K., Sarwar M.A., Mahmood A., Chen Z.-L., Li Q., Zeng X.-P., Liu Y., Li Y.-R. (2020). Fate of nitrogen in agriculture and environment: Agronomic, eco-physiological and molecular approaches to improve nitrogen use efficiency. Biol. Res..

[43] Liu Q., Wu K., Song W., Zhong N., Wu Y., Fu X. (2022). Improving crop nitrogen use efficiency toward sustainable green revolution. Annu. Rev. Plant Biol..

[44] Kapoor P., Dhaka R.K., Sihag P., Mehla S., Sagwal V., Singh Y., Sonu L., Priyanka B., Krishna Pal S., Baoshan X. (2022). Nanotechnology-enabled biofortification strategies for micronutrients enrichment of food crops: Current understanding and future scope. NanoImpact.

[45] Shang Y., Hasan M.K., Ahammed G.J., Li M., Yin H., Zhou J. (2019). Applications of Nanotechnology in Plant Growth and Crop Protection: A Review. Molecules.

[46] Baig N., Kammakakam I., Falath W. (2021). Nanomaterials: A review of synthesis methods, properties, recent progress, and challenges. Mater. Adv..

[47] Koca F.D., Halici M.G., Işik Y., Ünal G. (2022). Green synthesis of Ag-ZnO nanocomposites by using Usnea florida and Pseudevernia furfuracea lichen extracts and evaluation of their neurotoxic effects. Inorg. Nano-Met. Chem..

[48] Porrawatkul P., Pimsen R., Kuyyogsuy A., Teppaya N., Noypha A., Chanthai S., Nuengmatcha P. (2022). Microwave-assisted synthesis of Ag/ZnO nanoparticles using Averrhoa carambola fruit extract as the reducing agent and their application in cotton fabrics with antibacterial and UV-protection properties. RSC Adv..

[49] Hameed S., Khalil A.T., Ali M., Numan M., Khamlich S., Shinwari Z.K., Maaza M. (2019). Greener synthesis of ZnO and Ag–ZnO nanoparticles using Silybum marianum for diverse biomedical applications. Nanomedicine.

[50] El Shafey A.M. (2020). Green synthesis of metal and metal oxide nanoparticles from plant leaf extracts and their applications: A review. Green Processing Synth..

[51] Gour A., Jain N.K. (2019). Advances in green synthesis of nanoparticles. Artif. Cells Nanomed. Biotechnol..

[52] Ahmed S.F., Mofijur M., Rafa N., Chowdhury A.T., Chowdhury S., Nahrin M., Islam A.S., Ong H.C. (2022). Green approaches in synthesising nanomaterials for environmental nanobioremediation: Technological advancements, applications, benefits and challenges. Environ. Res..

[53] Thirumalai K., Shanthi M., Swaminathan M. (2017). Natural sunlight active GdVO4–ZnO nanomaterials for photo–electrocatalytic and self–cleaning applications. J. Water Process Eng..

[54] Hamidian K., Sarani M., Barani M., Khakbaz F. (2022). Cytotoxic performance of green synthesized Ag and Mg dual doped ZnO NPs using Salvadora persica extract against MDA-MB-231 and MCF-10 cells. Arab. J. Chem..

[55] Sharwani A.A., Narayanan K.B., Khan M.E., Han S.S. (2022). Photocatalytic degradation activity of goji berry extract synthesized silver-loaded mesoporous zinc oxide (Ag@ ZnO) nanocomposites under simulated solar light irradiation. Sci. Rep..

[56] Jha P.K., Chawengkijwanich C., Techato K., Limbut W., Luengchavanon M. (2022). Callistemon viminalis Leaf Extract Mediated Biosynthesis of Ag, rGO-Ag-ZnO Nanomaterials for Catalytic PEM Fuel Cell Application. Trends Sci..

[57] Guo Y., Fu X., Liu R., Chu M., Tian W. (2022). Efficient green photocatalyst of Ag/ZnO nanoparticles for methylene blue photodegradation. J. Mater. Sci. Mater. Electron..

[58] Dikshit P.K., Kumar J., Das A.K., Sadhu S., Sharma S., Singh S., Gupta P.K., Kim B.S. (2021). Green synthesis of metallic nanoparticles: Applications and limitations. Catalysts.

[59] Imtiaz H., Shiraz M., Mir A.R., Hayat S. (2022). Green synthesis of nanoparticles and their effect on plant growth and development: A review. Plant Arch..

[60] Naz R., Aftab M., Sarwar G., Aslam A., Nazir Q., Naz A., Niaz A., Rasheed F., Kalsom A., Mukhtar N. (2022). Evaluation of temporal and differential fertilizer application on growth, yield and quality of wheat. Pak. J. Agric. Res..

[61] Landa P. (2021). Positive effects of metallic nanoparticles on plants: Overview of involved mechanisms. Plant Physiol. Biochem..

[62] Salih A.M., Qahtan A.A., Al-Qurainy F., Al-Munqedhi B.M. (2022). Impact of Biogenic Ag-Containing Nanoparticles on Germination Rate, Growth, Physiological, Biochemical Parameters, and Antioxidants System of Tomato (*Solanum tuberosum* L.) In Vitro. Processes.

[63] Ali A., Mohammad S., Khan M.A., Raja N.I., Arif M., Kamil A., Mashwani Z.U.R. (2019). Silver nanoparticles elicited in vitro callus cultures for accumulation of biomass and secondary metabolites in Caralluma tuberculata. Artif. Cells Nanomed. Biotechnol..

[64] Ehsan M., Waheed A., Ullah A., Kazmi A., Ali A., Raja N.I., Mashwani Z.-U., Sultana T., Mustafa N., Ikram M. (2022). Plant-Based Bimetallic Silver-Zinc Oxide Nanoparticles: A Comprehensive Perspective of Synthesis, Biomedical Applications, and Future Trends. BioMed Res. Int..

[65] Hasanuzzaman M., Bhuyan M.H.M., Zulfiqar F., Raza A., Mohsin S.M., Mahmud J.A., Fujita M., Fotopoulos V. (2020). Reactive oxygen species and antioxidant defense in plants under abiotic stress: Revisiting the crucial role of a universal defense regulator. Antioxidants.

[66] Farooq T., Nisa Z.U., Hameed A., Ahmed T., Hameed A. (2022). Priming with copper-chitosan nanoparticles elicit tolerance against PEG-induced hyperosmotic stress and salinity in wheat. BMC Chem..

[67] Alabdallah N.M., Hasan M.M., Hammami I., Alghamdi A.I., Alshehri D., Alatawi H.A. (2021). Green synthesized metal oxide nanoparticles mediate growth regulation and physiology of crop plants under drought stress. Plants.

[68] Lahuta L.B., Szablińska-Piernik J., Głowacka K., Stałanowska K., Railean-Plugaru V., Horbowicz M., Pomastowski P., Buszewski B. (2022). The Effect of Bio-Synthesized Silver Nanoparticles on Germination, Early Seedling Development, and Metabolome of Wheat (*Triticum aestivum* L.). Molecules.

[69] Darjee S., Shrivastava M., Langyan S., Singh G., Pandey R., Sharma A., Khandelwal A., Singh R. (2022). Integrated nutrient management reduced the nutrient losses and increased crop yield in irrigated wheat. Arch. Agron. Soil Sci..

[70] Xu X., Ma F., Zhou J., Du C. (2022). Control-released urea improved agricultural production efficiency and reduced the ecological and environmental impact in rice-wheat rotation system: A life-cycle perspective. Field Crops Res..

